# Clinical applicability of biologically effective dose calculation for spinal cord in fractionated spine stereotactic body radiation therapy

**DOI:** 10.1515/raon-2015-0008

**Published:** 2015-03-25

**Authors:** Seung Heon Lee, Kyu Chan Lee, Jinho Choi, So Hyun Ahn, Seok Ho Lee, Ki Hoon Sung, Se Hee Kil

**Affiliations:** 1 Department of Radiation Oncology; Gachon University Gil Medical Center, Republic of Korea; 2 Gachon Medical Research Institute, Gachon University Gil Medical Center, Republic of Korea

**Keywords:** biologically effective dose, spine stereotactic body radiation therapy, spinal cord, tolerance dose, linear quadratic model

## Abstract

**Background.:**

The aim of the study was to investigate whether biologically effective dose (BED) based on linear-quadratic model can be used to estimate spinal cord tolerance dose in spine stereotactic body radiation therapy (SBRT) delivered in 4 or more fractions.

**Patients and methods.:**

Sixty-three metastatic spinal lesions in 47 patients were retrospectively evaluated. The most frequently prescribed dose was 36 Gy in 4 fractions. In planning, we tried to limit the maximum dose to the spinal cord or *cauda equina* less than 50% of prescription or 45 Gy_2/2_. BED was calculated using maximum point dose of spinal cord.

**Results.:**

Maximum spinal cord dose per fraction ranged from 2.6 to 6.0 Gy (median 4.3 Gy). Except 4 patients with 52.7, 56.4, 62.4, and 67.9 Gy_2/2_, equivalent total dose in 2-Gy fraction of the patients was not more than 50 Gy_2/2_ (12.1–67.9, median 32.0). The ratio of maximum spinal cord dose to prescription dose increased up to 82.2% of prescription dose as epidural spinal cord compression grade increased. No patient developed grade 2 or higher radiation-induced spinal cord toxicity during follow-up period of 0.5 to 53.9 months.

**Conclusions.:**

In fractionated spine SBRT, BED can be used to estimate spinal cord tolerance dose, provided that the dose per fraction to the spinal cord is moderate, e.g. < 6.0 Gy. It appears that a maximum dose of up to 45–50 Gy_2/2_ to the spinal cord is tolerable in 4 or more fractionation regimen.

## Introduction

Stereotactic body radiation therapy (SBRT) has been increasingly applied to the management of spinal metastases with encouraging clinical results of rapid and durable pain relief.^1^,^2^ SBRT is also effective for treating radio-resistant metastatic tumours, such as, renal cell carcinoma or malignant melanoma.^3^,^4^

The spinal cord is the major dose-limiting tissue in spine SBRT. In conventionally fractionated radiation therapy, the tolerance dose for the spinal cord has been reported to be 50 Gy for cord lengths of 5 and 10 cm, and 47 Gy for 20 cm, given a probability of myelopathy of less than 5% within 5 years.^5^ Schultheiss reported that the probabilities of myelopathy were 0.03% and 0.2% at 45 Gy and 50 Gy, respectively.^6^ For single fraction SBRT, Ryu *et al.* reported a partial volume tolerance of the human spinal cord of at least 10 Gy to 10% of the spinal cord volume when spinal cord volume was defined from 6 mm above and to 6 mm below the treatment target.^7^ Sahgal *et al.* suggested 10 Gy as a maximum safe threshold for single fraction SBRT to the thecal sac.^8^

In fractionated spine SBRT, however, variable dose schedules are applied and no reliable dose comparison method has been established for the target or the spinal cord. Since Fowler first proposed the term ‘biologically effective dose’ based on linear-quadratic (LQ) cell survival model in 1989, BED has been used to compare the biologic effects of various radiotherapy schedules.^9^ However, because prescription doses in fractionated spine SBRT are usually between 6 and 10 Gy per fraction, several authors have argued that the simple application of BED based on LQ model is not appropriate in SBRT.^10^–^18^ The extrapolations using the LQ model beyond 5–6 Gy per fraction are likely to lack clinically useful precision.^19^

Modern linear accelerator based stereotactic radiotherapy technology using a fine multileaf collimator of 2.5 mm thickness could deliver highly conformal radiation to the target while sparing the spinal cord with the merit of a steep dose gradient just outside the target. The irradiated dose to the spinal cord can be more strictly limited and is usually much lower than the prescription dose in fractionated spine SBRT. We hypothesized that if maximum doses per fraction to the spinal cord are less than 6 Gy, BED based on LQ model could be used to estimate spinal cord tolerance dose in fractionated spine SBRT. We usually implemented fractionated spine SBRT in 4 or 5 fractions to avoid complications such as radiation-induced myelopathy and vertebral compression fracture.

To determine if BED based on LQ model can be used to estimate spinal cord tolerance dose in fractionated spine SBRT of 4 or more fractionation regimen, the plans used for actual fractionated SBRT at our institution were analysed retrospectively and clinical outcomes, including complications, were investigated.

## Patients and methods

Sixty-three metastatic spinal lesions in 47 patients were treated by spine SBRT between January 2010 and March 2014. Median patient age was 56 (range 33–86) and 27 patients (57.4%) were male. The thoracic spine was the most frequent site for treatment. Of the 63 lesions, 41 lesions involved single or two contiguous spine segments. When categorized with the epidural spinal cord compression (ESCC) grading system, 43.5% of the lesions belonged to grade 1c (deformation of the thecal sac with spinal cord abutment), 2 (spinal cord compression, but with cerebrospinal fluid [CSF] visible around the cord) or 3 (spinal cord compression, no CSF visible around the cord) (Table 1).^20^ No patients received surgical intervention, vertebroplasty or kyphoplasty, prior to spine SBRT.

All patients underwent CT simulation with an appropriate immobilization technique to obtain SBRT planning images. To delineate targets and spinal cords, T2-weighted and gadolinium contrast T1-weighted MRI sequences with a 3 mm slice thickness including at least one vertebral body above and below the target were obtained and fused to the planning CT image using iPlan software (version 4.1, BrainLAB, Germany). Gross tumour volume (GTV) included gross visible tumour in spine, paraspinal, or epidural area on MR or enhanced planning CT images. Planning target volumes (PTV) were derived from GTVs by encompassing involved vertebral body and including anterior and/or posterior elements of the spine depending on the location of metastatic lesions as described in RTOG 0631.^21^ Median PTV volume was 65.6 cc (range, 17.9–340.8 cc). The spinal cord was contoured starting from 6 mm above the superior of the PTV to 6 mm below the inferior of the PTV. However, the spinal cord was always excluded from the PTV, with a 1–2 mm free margin if the GTV did not abut onto the cord. Other organs, such as heart, lungs, oesophagus, large vessels, trachea, liver, and kidneys were delineated depending on tumour vertebral level.

The most frequently prescribed dose was 36 Gy in 4 fractions, followed by 40 Gy in 5 fractions (Table 2). The requirement for clinical implementation was > 80% of the prescription dose to > 90% of the PTV, or a mean PTV dose > 95% of prescription. The spinal cord dose was converted to equivalent total dose in 2-Gy fraction (EQD_2_) using the following formula provided below. This model was derived from the LQ model assuming an α/β ratio of 2 for the late effect of spinal cord.

$${\text{EQD}}_{2}\left({\text{GY}}_{2/2}\right)=\text{Total}\;\text{dose}\times \left(\frac{\text{Dose}\;\text{per}\;\text{fraction}+\alpha /\beta }{2+\alpha /\beta }\right)$$

We tried to limit the maximum dose to the spinal cord or *cauda equina* less than 50% of prescription or 45 Gy_2/2_. Maximum dose per fraction to the spinal cord of each plan was investigated.

All patients were treated using the Novalis Tx^TM^ (Varian, USA) equipped with a 2.5 mm multileaf collimator. Thirty-five patients received SBRT to a single treatment site spanning one to five vertebral segments. Eight patients received SBRT to 2 separate sites and 4 patients to 3 sites. Re-SBRT was performed in one patient with hepatocellular carcinoma at 5 months after initial SBRT because of the recurrence of severe pain due to tumour progression.

Patients were followed up clinically and radiographically at 1- to 3-month intervals. A visual analogue scale (VAS) was used to measure pain before and after treatment. Symptomatic responses were scored as defined by RTOG 0631.^21^ All available follow up MRIs were reviewed to assess radiographic responses. Radiologic local failure was defined as local tumour growth by MRI. Late complications were scored as described by Common Toxicity Criteria for Adverse Events, version 4.0. Overall survival was estimated by the Kaplan–Meier method. The relationship between radiologic local failure and variable candidate risk factors such as spinal cord to tumour distance, spinal cord to PTV distance, minimum dose administered to tumour, and tumour volume was analyzed by the Mann-Whitney U-test. The retrospective study was approved by the institutional review board committee and was according to the Helsinki Declaration.

## Results

**FIGURE 1.**
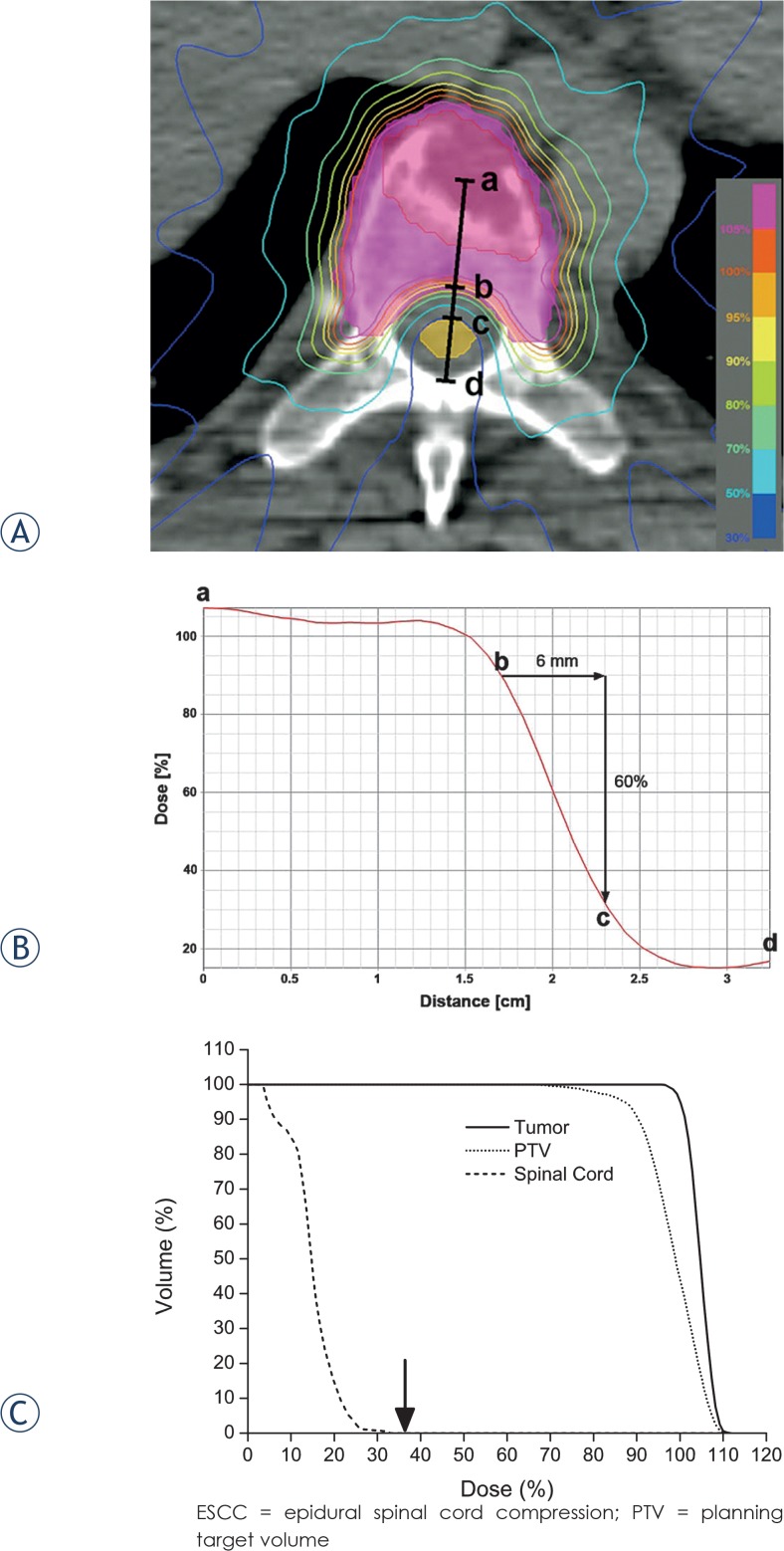
An example of dose distribution in a patient with disease of the T7 vertebral body only (ESCC grade^20^ 0). **(A, B)** Dose profile from the center of the tumour (a) to the posterior edge of the thecal sac (d). Note that the dose gradient around the spinal cord in this case is steepest between (b) and (c), where 90% and 30% isodose lines, and is approximately 10% per millimeter. **(C)** Dose volume histogram of the patient shows much difference of the doses to target and spinal cord. The maximum dose per fraction to the spinal cord (arrow) was 37% (3.0 Gy) of the prescription dose (9.0 Gy).

Inhomogeneous dose distributions inside the spinal cord and very steep dose gradients around it were observed (Figure 1A). Approximately 10% decrease of dose per millimetre was observed from PTV margin to the surface of spinal cord (Figure 1B). Maximum dose to spinal cord was much lower than prescription dose (Figure 1C). According to PTV shape and spinal cord proximity, maximum spinal cord doses varied from 10.5 Gy to 33.2 Gy (median 20.2 Gy). When they were divided by corresponding fraction numbers, the maximum spinal cord dose per fraction ranged from 2.6 to 6.0 Gy (median 4.3 Gy). Maximum EQD_2_ to spinal cords ranged from 12.1 to 67.9 Gy_2/2_ (median 32.0 Gy_2/2_). Six cords were administered more than 45 Gy_2/2_ and doses of them were 48.0, 49.0, 52.7, 56.4, 62.4, and 67.9 Gy_2/2_, respectively. The ratio of maximum spinal cord dose to prescription dose increased up to 82.2% of prescription dose as the ESCC grade increased (Table 3).

Median follow-up period was 7.1 months (0.5–53.9 months) and median overall survival was 10.2 months. During follow-up, 26 patients succumbed to systemic disease progression. VAS results were available for 46 of the 63 lesions. Mean VAS declined from 7.8 before to 2.7 after SBRT. Complete response was achieved for 10 lesions and partial response for 28 lesions.

Follow-up MRIs were available for 27 lesions. Radiologic local failure occurred in 4 lesions (14.8%, Table 4). All patients in the radiologic local failure group (4 patients) had spinal cord compression (ESCC grade 2) before SBRT. In the non-failure group (23 patients), distances between spinal cords and tumours or PTVs ranged from 0 to 14 mm (median 2.3) or from 0 to 3.5 mm (median 1.0), respectively. Spinal cord to tumour distance (p < 0.001), spinal cord to PTV distance (p = 0.003), and minimum dose administered to tumours (p = 0.040) in the local failure group were significantly smaller than those in the non-failure group. No intergroup difference of tumour volumes was observed. There has been no grade 2 or more radiation-induced spinal cord toxicity during follow up period up to 53.9 months. There were three compression fractures (3 of 27, 11.1%); two resulted from progressions of existing fractures and the other was a new fracture. Fractures occurred at 2, 3, and 4 months after treatment, respectively.

## Discussion

BED based on the LQ model has been widely accepted for the comparisons of doses administered in different treatment schedules in treating with conventional multifractionated irradiation. However, dose comparisons for fractionated spine SBRT based on simple BED calculations should be approached with caution. First, BED was developed and validated based on homogenous radiation dose distributions in irradiated areas.^22^,^23^ The radiation dose administered to the spinal cord in SBRT is intrinsically inhomogeneous because it is performed with an intensity modulated radiation beam. The data regarding the tolerance of the rat cervical spinal cord suggested that small volume of rat spinal cord tolerates a greater dose compared to homogeneous radiation.^24^,^25^ Although those observations were not found in swine model, the tolerance dose of spinal cord for partial-volume irradiation closely resembled that for rats, mice and guinea pigs receiving uniform spinal cord irradiation.^26^ Therefore, estimated spinal cord tolerance using BED calculation for partial-volume irradiation seems to be more conservative than, or at least comparable to that for uniform irradiation.

Second, BED has been based on the data of conventional fraction size. However, in fractionated SBRT, prescription dose per fraction is relatively high, usually 6–20 Gy. Fundamental arguments have arisen as to whether the LQ model is a valid method for assessing BED when doses per fraction are high. Brenner *et al*. reported that the LQ model is reasonably well validated experimentally and theoretically up to about 10 Gy per fraction, and suggested that its use is reasonable up to about 18 Gy per fraction.^27^ However, several authors have argued to the contrary. Iwata *et al.* studied the applicability of the LQ model for dose conversion in high dose per fraction radiotherapy using cell survival data for V79 Chinese hamster lung fibro-blasts and EMT6 mouse mammary sarcoma cells.^14^ It was found that the LQ model fitted relatively well at doses of 5 Gy or less as compared with the repairable-conditionally repairable model and the multi-target model. Timmerman *et al*. proposed a universal survival curve that hybridizes the LQ model survival curve for the low-dose range and the multi-target model asymptote for the high-dose range.^15^ They reported a transition dose at which the LQ model smoothly transits to the terminal asymptote of the multi-target model. The transition dose calculated using 12 non-small-cell lung cancer (NSCLC) cell lines was 6.2 Gy, which means that LQ model may not be applicable for dose ranges of more than 6.2 Gy. Recently, Song *et al.* argued that the usefulness of the LQ model is likely to be limited when tumours are treated with high dose per fraction, usually more than 10 Gy, because LQ model and other modified-LQ models are based on the assumption that radiation-induced cell death in tumours is due solely to DNA strand breaks. They suggested indirect/necrotic cell death as a consequence of vascular damage plays an important role in SBRT.^17^,^18^ As of now, the applicability of BED based on the LQ model to the high dose per fraction radiation remains a controversial issue.

According to the current radiobiological knowledge as mentioned above, BED based on the LQ model seems to be clinically applicable if the dose is limited to 6 Gy or less, especially for normal tissue, not tumour. With current technical developments, dose to the spinal cord can be maintained at much lower levels than the prescription dose due to the steep dose gradient just outside the target. In the present study, the maximum irradiation dose per fraction to the spinal cord varied from 2.6 to 6.0 Gy (median 4.3 Gy) depending on the PTV shape and its proximity to the spinal cord. Because the dose per fraction to the spinal cord was less than 6.0 Gy, it would be reasonable to estimate spinal cord tolerance dose in fractionated SBRT using BED based on LQ model.

Recently, Sahgal *et al.* recommended limiting maximum point dose to 23.0 Gy in 4 fractions and 25.3 Gy in 5 fractions for a risk of radiation myelopathy of less than 5%.^28^ Dividing the constraint dose by fraction number, maximum point doses per fraction to the thecal sac are 5.75 Gy and 5.06 Gy, respectively. The calculated EQD_2_ of thecal sac based on the LQ model for these schedules were 44.6 Gy_2/2_ and 44.7 Gy_2/2_, respectively. These seem to be reasonable because the tolerance dose of spinal cord in conventionally fractionated radiation therapy is 45-50 Gy with fraction size of 1.8 or 2.0 Gy when full thickness of the cord is irradiated. In Sahgal’s data, there was no radiation myelitis after irradiation in 4 or 5 fractions, though the patient number is relatively small, 9 cases. In the present study, except 4 patients, all patients were administered a maximum spinal cord dose less than 50 Gy_2/2_ and no radiation myelopathy was observed among 63 cases.

Gerszten *et al*. reported that post-SBRT tumour progressions often occurred at the edge of contoured treatment volumes and the overall mean tumour volume of local failure cases was 40% greater than the average for their series.^29^ Chang *et al.* also reported that failure at the epidural space adjacent to the spinal cord is a major reason for tumour progression after spine SBRT.^10^ In the present study, minimum tumour dose (p = 0.040), which is mainly affected by distance between spinal cord and tumour (p < 0.001) or PTV (p = 0.003) appeared to have more influence on local failure. Furthermore, when the tumour did not abut the spinal cord, local failure was not observed even in tumours larger than those in local failure group. These results mean that tumoricidal dose was not delivered to tumour because of the proximity of spinal cord in local failure group. BED calculation has clinical impact on choosing appropriate fraction size and number to deliver optimal tumour dose, especially for the lesions close to spinal cord, while limiting spinal cord dose of less than 45–50 Gy_2/2_.

**FIGURE 2.**
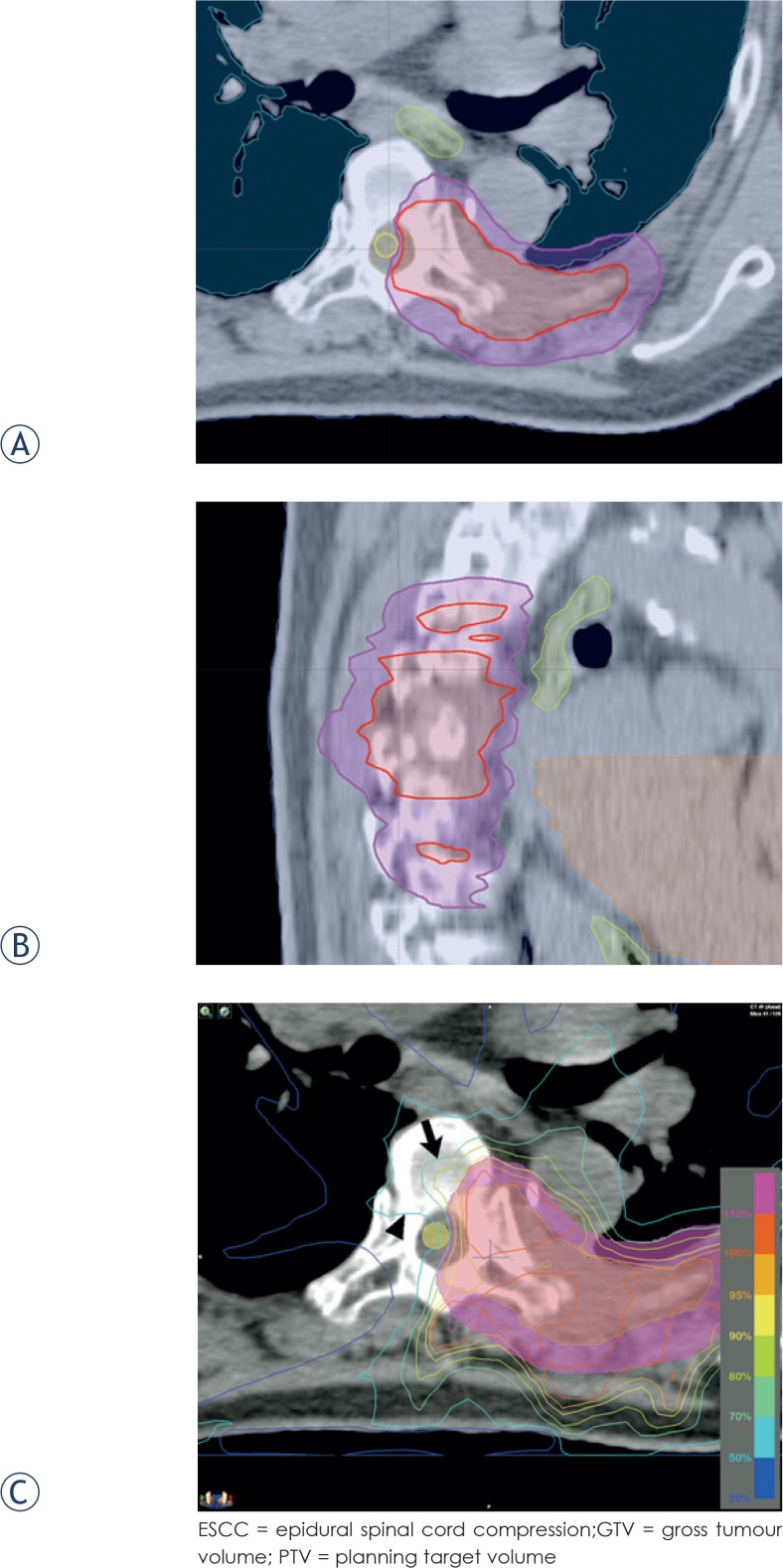
**(A, B)** Target volume (GTV: red, PTV: magenta) delineation in a patient with cord compression (ESCC grade^20^ 2) and paraspinal mass. Five spine segments were involved in the PTV. **(C)** Dose distribution around the spinal cord. The 70% (arrow) and 50% (arrowhead) isodose lines are shown. The maximum spinal cord dose was 33.2 Gy, which was equivalent to 78.9% of the prescription dose of 42 Gy in 6 fractions.

When a tumour abuts the spinal cord, increasing the number of fractions to deliver potentially tumoricidal dose, with lowering spinal cord BED, might be considered. In the patient shown in Figure 2, there was a large paraspinal tumour mass compressing the spinal cord. By increasing the number of fractions to 6 and decreasing the prescription dose per fraction to the PTV to 7 Gy, we tried to spare the spinal cord delivering 42 Gy to the GTV to improve local control. Although maximum EQD_2_ of the spinal cord was 62.4 Gy_2/2_, we treated this patient in the palliative setting because local tumour control was important for the quality of life of the patient.

This study has several limitations; it is a retrospective and single center study, cohort was small, and there was no myelopathy case. Rare but severe events like myelopathy require high patient numbers to evaluate safe tolerance doses and it should not give a false sense of security. Although no myelopathy was observed in 6 patients who were administered a maximum spinal cord dose greater than 45 Gy_2/2_, their survival period was only 1.5–6.4 months. They had multiple metastases in liver or lung and had short life expectancy. Therefore, it is not possible to suggest more doses for spinal cord tolerance above this dose level. However, we believe that the data of our study is important for applying BED calculation for spinal cord tolerance dose in various clinical situations. We plan to conduct a multi-center prospective study with more patients.

In conclusion, BED can be used to estimate spinal cord tolerance dose, provided that the dose per fraction to the spinal cord is moderate, e.g. < 6.0 Gy in fractionated spine SBRT. Within this dose range it appears that a maximum dose of up to 45–50 Gy_2/2_ to the spinal cord is tolerable. The minimum tumour dose, which is mainly affected by tumour to spinal cord distance, seems to significantly affect local failure. When a tumour abuts or is closely located to the spinal cord, we suggest adjustment of the fractionation schedule based on BED calculations, while maintaining the desirable dose to the target. Randomized controlled dose escalation study is reserved to verify this suggestion.

## Figures and Tables

**TABLE 1.** Patient and tumour characteristics

Age (year, median)	33–86 (56)
Histology (person)	
Lung	13
Colorectal	11
Breast	6
Pancreas	3
Hepatocellular	3
Stomach	2
Cholangiocarcinoma	2
Prostate	2
Renal cell	1
Other	4
Spine level (lesion)	
Cervical	3
Cervicothoracic	4
Thoracic	37
Thoracolumbar	5
Lumbar	14
Number of involved spine segments per PTV (lesion)	
1	24
2	17
3	16
4	3
5	3
Tumour volume (cc, median)	1.0–176.7 (21.0)
PTV volume (cc, median)	17.9–340.8 (59.1)
Number of treated sites per patient (person)	
1	35
2	8
3	4
ESCC grade^20^ (lesion)	
0	14
1a	15
1b	9
1c	5
2	18
3	2

ESCC = epidural spinal cord compression; PTV = planning target volume

**TABLE 2.** Prescription dose to planning target volume and maximum dose to spinal cord

**Total dose / fractions**	**Number**	**Maximum dose to spinal cord (EQD_2_, Gy_2/2_)**
26.0 Gy / 4 fractions	1	24.8
28.0 Gy / 4 fractions	1	25.4
30.0 Gy / 4 fractions	1	52.7
32.0 Gy / 4 fractions	2	25.5–56.4
36.0 Gy / 4 fractions	23	12.1–67.9
40.0 Gy / 4 fractions	1	24.3
44.0 Gy / 4 fractions	2	38.9–44.4
32.5 Gy / 5 fractions	6	16.6–43.0
35.0 Gy / 5 fractions	4	25.7–49.0
40.0 Gy / 5 fractions	19	24.2–41.1
42.5 Gy / 5 fractions	1	38.7
42.0 Gy / 6 fractions	2	39.8–62.4

BED = biologically effective dose; EQD_2_ = equivalent dose in 2-Gy fractions with an α/β ratio of 2

**TABLE 3.** Spinal cord dose classified using the epidural spinal cord compression (ESCC) grading system^20^

**Grade**	**lesions**	**D_max_ (Gy, median)**	**EQD_2 max_ (Gy_2/2_, median)**	**D_max_ / prescription dose (%, median)**
0	14	12.0–25.5 (18.3)	15*.0*–*38*.9 (29.6)	33.4–60.7 (50.5)
1a	15	10.5–25.3 (20.8)	12.1–52.7 (32.0)	29.1–84.3 (52.0)
1b	9	13.9–23.4 (19.3)	16.6–44.4 (33.4)	40.3–56.2 (53.4)
1c	5	16.3–22.3 (17.5)	24.8–36.1 (28.0)	45.9–63.8 (57.1)
2	18	16.5–33.2 (21.8)	24.2–67.9 (36.1)	43.9–81.1 (58.4)
3	2	21.4–26.3 (23.9)	39.3–56.4 (47.9)	59.4–82.2 (70.8)

D_max_ = maximum dose to spinal cord; EQD_2max_ = maximum equivalent dose in 2-Gy fractions with an α/β ratio of 2; ESCC = epidural spinal cord compression

**TABLE 4.** Distances from spinal cord to tumour or planning target volume (PTV), minimum tumour doses, and tumour volumes according to radiologic local failure status

	**Failure group (4 lesions)**	**Non-failure group (23 lesions)**	p-value**
Distance between SC and tumour (mm, median)	0*	0–14.0 (2.3)	< 0.001
Distance between SC and PTV (mm, median)	0*	0–3.5 (1.0)	0.003
Minimum tumour dose (Gy, median)	15.4–23.8 (20.0)	15.3–44.7 (25.2)	0.040
Tumour volume (cc, median)	13.7–47.3 (20.8)	1.0–176.7 (20.9)	0.468

SC = spinal cord;

* All tumours compressed the spinal cord;

** Statistical significance was determined using the Mann-Whitney U-test
